# Digital evolution: enhancing exercise-based cardiac rehabilitation in the post-operative era

**DOI:** 10.1097/JS9.0000000000003515

**Published:** 2025-09-09

**Authors:** Bin Cao, Binning Ma, Zhenxing Deng

**Affiliations:** a Department of Cardiology, The Central Hospital of Yongzhou, Yongzhou, China; b Department of Cardiology, Yongzhou Hospital Affiliated to University of South China, Yongzhou, China

*Dear editor,*

We read with great interest the systematic review and meta-analysis by Shi entitled “Exercise-based cardiac rehabilitation for patients undergoing coronary artery operation: a systematic review and meta-analysis based on current randomized controlled trials” recently published in the International Journal of Surgery[1]. The authors provide a valuable synthesis of evidence confirming the significant benefits of exercise-based cardiac rehabilitation (CR) in improving health-related quality of life (HRQoL) and reducing myocardial infarction (MI) incidence and all-cause hospital admissions for post-CABG and post-PCI patients, without increasing mortality or complication rates. This robust analysis is a timely contribution to the field.

While the findings solidify the role of conventional exercise-based CR, we suggest the rapidly evolving landscape of digital health and artificial intelligence (AI) presents an unprecedented opportunity to enhance and extend these documented benefits. The conclusion regarding the significant HRQoL improvement (SMD: 0.24 for SF-36 summary; SMD: 0.35 for SF-36 domains) and reduction in hospital admissions (RR: 0.74) offers a compelling platform to explore how integrating advanced technologies could further optimize personalized rehabilitation pathways and potentially maximize these outcomes. We wish to briefly elaborate on this perspective, building logically upon the core findings.

Firstly, AI-driven analytics can transform standardized CR protocols into highly personalized prescriptions[2]. Machine learning models, trained on multimodal datasets including patient demographics, detailed surgical history (e.g., specific graft types in CABG, stent characteristics in PCI), comorbidities, genomic data, continuous physiological monitoring, and even patient-reported outcomes captured digitally, can predict individual responses to specific exercise intensities, modalities, and progression rates more accurately than traditional risk stratification alone. The meta-analysis underscores the effectiveness of exercise-CR broadly; AI personalization could potentially amplify the magnitude of HRQoL gains and further reduce hospital readmissions by preventing adverse events related to sub-optimal exercise dosing and optimizing adherence, a key factor in sustaining benefits. Furthermore, such models could identify patients most likely to benefit from specific interventions highlighted as effective in the analysis (like the pronounced MI reduction, RR: 0.50), allowing for more targeted resource allocation.

Secondly, digital health technologies, particularly remote patient monitoring (RPM) using wearable sensors (accelerometers, heart rate monitors, electrocardiogram patches), enable continuous, real-time objective assessment of patient activity levels, physiological responses, and adherence outside the supervised clinical environment[3]. This rich, longitudinal data stream is crucial for safe progression and early detection of potential complications. The significantly reduced hospital admission rate associated with exercise-CR in the findings could potentially be further enhanced by RPM. Early algorithmic detection of concerning physiological trends (e.g., arrhythmias, excessive fatigue signatures, ischemic ST-segment changes triggered by activity) via continuous monitoring could trigger timely clinical intervention, potentially preventing readmissions linked to undetected deterioration. RPM also provides granular adherence data far superior to self-report, enabling targeted support for patients struggling with engagement.

Thirdly, the concept of the “digital twin,” a dynamic, virtual model of an individual patient informed by real-time data streams, offers a paradigm shift towards ultra-personalized medicine and predictive rehabilitation^[4–6]^. Integrating data from wearables, electronic health records (including specific surgical details relevant to recovery kinetics), genomic profiles, and even patient-reported outcomes creates a comprehensive digital representation. Computational models can then simulate the potential impact of different CR strategies on this virtual twin before implementing them on the actual patient. This allows clinicians to virtually “test” and optimize exercise prescriptions, predict potential adverse responses (thus mitigating risk), and explore scenarios to maximize HRQoL and functional recovery outcomes specific to that individual, based directly on the efficacy parameters the meta-analysis robustly demonstrated. This predictive capability could fundamentally refine implementation protocols.

Successfully translating these concepts requires overcoming challenges related to data integration, algorithm validation, robust cybersecurity, equitable access, and defining clinical and economic value through rigorous implementation science frameworks[7]. However, the clear efficacy foundation established in analyses provides a strong justification for investigating how AI and digital health can be leveraged not to replace, but to empower and optimize traditional exercise-based CR. Future research priorities should include pragmatic trials integrating these technologies into post-coronary artery surgery rehabilitation pathways, explicitly measuring their impact on enhancing the core outcomes – HRQoL, mortality, MI, and hospitalization rates – the valuable study has confirmed. (We ensure this article is compliant with the TITAN Guidelines[8].)

## Data Availability

Not applicable.
